# Effect of Different Post-space Pretreatment Agents on the Push-Out Bond Strength of Fibre Posts Cemented With Self-Adhesive Resin Cement: An In Vitro Study

**DOI:** 10.7759/cureus.113874

**Published:** 2026-08-03

**Authors:** Shalini Kumari, Sufia Parveen, Chi Koy Wang, Abhinav Kumar Singh, Somnath Pal, Binoy Kumar Singh, Amit Kumar

**Affiliations:** 1 Department of Conservative Dentistry and Endodontics, Buddha Institute of Dental Sciences and Hospital, Patna, IND; 2 Department of Dermatology, Madhubani Medical College and Hospital, Madhubani, IND

**Keywords:** edta, endodontically treated teeth, glass fibre post, phosphoric acid, post-space pretreatment, push-out bond strength, radicular dentin, self-adhesive resin cement

## Abstract

Background: The long-term success of fibre post restorations is multifactorial and depends on several biomechanical and restorative factors, including the ferrule effect, the amount of remaining coronal tooth structure, restoration design, occlusal loading, and the quality of adhesion between the fibre post, resin cement, and radicular dentin. Among these factors, durable adhesive bonding is considered essential for enhancing post retention and the longevity of the restoration. The smear layer created during post-space preparation may compromise this adhesion by limiting resin infiltration. Pretreatment of the post space with different conditioning agents has been proposed to improve the bonding performance of self-adhesive resin cements; however, comparative evidence regarding these four conditioning agents remains limited. The present study aimed to evaluate and compare the effect of different post-space pretreatment agents on the push-out bond strength of glass fibre posts luted with self-adhesive resin cement.

Materials and methods: Forty extracted human permanent maxillary central incisors were endodontically treated and randomly allocated into four groups (n = 10) according to the post-space pretreatment agent used: Group I (17% ethylenediaminetetraacetic acid (EDTA)), Group II (10% citric acid), Group III (10% polyacrylic acid), and Group IV (37% phosphoric acid). Following standardised post-space preparation, glass fibre posts were cemented using a self-adhesive resin cement. Each specimen was sectioned into 2-mm-thick slices representing the coronal, middle, and apical thirds of the root. Push-out bond strength was evaluated using a universal testing machine and expressed in megapascals (MPa). Data were analysed using one-way analysis of variance (ANOVA) followed by Tukey's honestly significant difference (HSD) post-hoc test for intergroup comparisons, and a linear mixed-effects model (REML) followed by Tukey's multiple comparisons test for within-group comparisons among the coronal, middle, and apical root thirds. A P-value < 0.05 was considered statistically significant.

Results: The 37% phosphoric acid group demonstrated the highest push-out bond strength at all root levels, followed by 10% polyacrylic acid, 10% citric acid, and 17% EDTA, which consistently showed the lowest values. Bond strength progressively decreased from the coronal to the apical third in all pretreatment groups. One-way ANOVA revealed significant intergroup differences at the coronal, middle, and apical root levels (all *P* < 0.0001). Tukey's HSD post hoc analysis demonstrated significant pairwise differences between most groups, except between the 10% citric acid and 10% polyacrylic acid groups at the middle and apical thirds. Linear mixed-effects model analysis showed significant differences in bond strength among the coronal, middle, and apical thirds within each pretreatment group (all *P* < 0.0001), with the greatest reduction occurring between the coronal and apical thirds.

Conclusion: Post-space pretreatment significantly influenced the immediate push-out bond strength of glass fibre posts cemented with self-adhesive resin cement. Among the conditioning agents evaluated, 37% phosphoric acid demonstrated the highest bond strength. Further in vivo studies are needed to confirm the clinical applicability of these findings.

## Introduction

Endodontically treated teeth frequently exhibit substantial loss of coronal tooth structure as a consequence of caries, trauma, previous restorations, or endodontic access preparation. Although root canal treatment effectively eliminates infection and preserves the tooth, the remaining dental structure is often biomechanically compromised, increasing the susceptibility to fracture under functional loading. Consequently, restoration of these teeth requires an approach that not only replaces the lost coronal structure but also provides adequate retention and long-term biomechanical stability [1].

In cases where insufficient coronal dentin remains to retain a core restoration, the use of an intraradicular post becomes essential. Contrary to the traditional belief that posts reinforce endodontically treated teeth, current evidence indicates that their principal function is to retain the core and support the definitive restoration rather than strengthen the root itself [2]. The longevity of post-retained restorations depends on several factors, including the amount of remaining tooth structure, post design, luting material, dentin characteristics, and the quality of adhesion achieved between the post, resin cement, and radicular dentin [3].

Metal posts have been extensively used because of their high strength and rigidity; however, their high elastic modulus differs considerably from that of dentin, resulting in unfavourable stress concentration within the root and an increased risk of catastrophic root fracture. In addition, metallic posts compromise esthetics and require greater removal of radicular dentin during post-space preparation [4]. These limitations have led to the widespread use of glass fibre posts, whose elastic modulus closely approximates that of dentin, enabling more uniform stress distribution and a lower incidence of irreparable root fractures. Furthermore, glass fibre posts provide superior esthetics, excellent biocompatibility, and improved compatibility with adhesive restorative systems, making them the preferred option for restoration of endodontically treated teeth, particularly in the esthetic zone [5].

Successful clinical performance of glass fibre posts depends largely on durable adhesion between the post, resin cement, and root canal dentin. During post-space preparation, instrumentation produces a smear layer composed of organic and inorganic debris, remnants of gutta-percha, sealer particles, and dentin fragments. This smear layer may occlude dentinal tubules and interfere with resin infiltration, thereby reducing micromechanical retention and compromising the bond strength of fibre posts. Therefore, effective modification or removal of the smear layer is considered a critical step for achieving predictable adhesive bonding within the root canal [6].

Various chemical conditioning agents have been investigated to optimise the dentin surface before fibre-post cementation. Ethylenediaminetetraacetic acid (EDTA) acts as a chelating agent that selectively removes the inorganic component of the smear layer without causing excessive demineralisation [7]. Citric acid is an organic acid capable of smear-layer removal and dentin demineralisation, whereas phosphoric acid functions as a strong etchant that effectively dissolves both the organic and inorganic components of the smear layer, exposing dentinal tubules and increasing dentin surface energy [8]. Polyacrylic acid, a mild conditioner commonly used in adhesive dentistry, partially removes the smear layer while preserving hydroxyapatite necessary for chemical interaction with resin-based materials. These differences in conditioning mechanisms may significantly influence the bonding performance of self-adhesive resin cements [9].

Self-adhesive resin cements have simplified the cementation procedure by eliminating separate etching, priming, and bonding steps. These materials contain multifunctional acidic monomers capable of simultaneously demineralising dentin and chemically interacting with hydroxyapatite, thereby reducing technique sensitivity and clinical chairside time. Nevertheless, the presence of a dense smear layer may limit resin penetration and reduce the effectiveness of both micromechanical interlocking and chemical bonding. Consequently, pretreatment of the post space before cementation has been proposed as a strategy to improve the bonding performance of self-adhesive resin cements [10].

The push-out bond strength test is considered one of the most reliable laboratory methods for evaluating the retention of fibre posts because the applied load closely simulates dislodging forces acting along the long axis of the post. However, like all in vitro tests, it cannot fully reproduce the complex intraoral environment, including cyclic occlusal loading, thermal changes, moisture, fatigue, and multidirectional functional stresses. Nevertheless, it remains a standardised and widely accepted method for comparing the bonding performance of different post-cementation protocols. In addition, this test enables separate evaluation of the coronal, middle, and apical thirds of the root canal, where differences in dentinal tubule density, smear-layer characteristics, accessibility, and resin polymerisation may influence adhesive performance [11].

Although several studies have independently investigated different root canal conditioning agents, the resin cement systems employed have varied considerably [12,13]. Previous investigations have used both conventional adhesive resin cement systems requiring separate etching and bonding procedures as well as self-adhesive resin cements, making direct comparison among studies difficult [9,13,14]. Consequently, the available evidence comparing 17% EDTA, 10% citric acid, 10% polyacrylic acid, and 37% phosphoric acid before cementation of glass fibre posts with a single self-adhesive resin cement remains scarce and inconclusive. Furthermore, few studies have compared their effects simultaneously at the coronal, middle, and apical levels of the root canal. Therefore, the present in vitro study was undertaken to evaluate and compare the effect of 17% EDTA, 10% citric acid, 10% polyacrylic acid, and 37% phosphoric acid as post-space pretreatment agents on the push-out bond strength of glass fibre posts cemented with self-adhesive resin cement.

## Materials and methods

Study design

The present in vitro experimental study was conducted in the Department of Conservative Dentistry and Endodontics, Buddha Institute of Dental Sciences and Hospital, Patna, Bihar, India. The study was designed to evaluate the effect of different post-space pretreatment agents on the push-out bond strength of glass fibre posts luted with self-adhesive resin cement. Ethical approval was obtained from the Institutional Ethics Committee of Buddha Institute of Dental Sciences and Hospital before commencement of the study (IEC Ref. No. 77/BIDSH/IEC/2024). Informed consent was obtained from patients prior to tooth collection.

Sample size calculation

The sample size was calculated a priori using G*Power software (version 3.1.9.7; Heinrich Heine University, Düsseldorf, Germany) for a fixed-effects one-way analysis of variance (ANOVA) comparing four independent groups. Based on a significance level (α) of 0.05, a statistical power (1−β) of 80%, and a large effect size (f = 0.50) derived from previous studies evaluating the push-out bond strength of fibre posts following different post-space pretreatment protocols, the minimum required sample size was estimated to be 36 specimens (nine specimens per group) [13]. To compensate for potential specimen loss during specimen preparation, sectioning, or mechanical testing, the sample size was increased to 40 specimens, with 10 specimens allocated to each experimental group.

Specimen selection

Forty freshly extracted human permanent maxillary central incisors were collected from the Department of Oral and Maxillofacial Surgery. Teeth extracted for reasons unrelated to the present investigation were included after obtaining informed consent from the patients.

Inclusion criteria

The study included freshly extracted human permanent maxillary central incisors with a single root and a single root canal. Only teeth with completely formed apices and intact root morphology were selected. The included teeth were free from dental caries, restorations, cracks, fractures, developmental anomalies, root resorption, or previous endodontic treatment. Radiographic examination confirmed the presence of a single canal without evidence of calcification, internal or external resorption, or aberrant canal morphology. Teeth exhibiting comparable root dimensions and minimal physiological curvature were selected to achieve standardisation of the experimental specimens and to reduce the influence of anatomical variability on bond strength measurements.

Exclusion criteria

Teeth with immature apices, multiple canals, severe root curvature, root fractures, craze lines, developmental defects, internal or external root resorption, canal obliteration, or calcification were excluded from the study. Specimens with previous endodontic treatment, post placement, restorations involving the root structure, extensive cervical abrasion, or structural damage occurring during extraction were also excluded. Teeth with excessive wear, anomalous root morphology, or any condition that could potentially affect the adhesion of the fibre post or interfere with the standardisation of the experimental procedures were not included in the study.

Specimen preparation

Following extraction, all soft tissue remnants and calculus deposits were removed using ultrasonic scalers. The teeth were disinfected by immersion in 0.5% sodium hypochlorite solution for 30 minutes and subsequently stored in 0.1% thymol solution (HiMedia Laboratories Pvt. Ltd., Mumbai, India) until use. Before endodontic treatment, radiovisiographic (RVG) examination was performed in both the mesiodistal and buccolingual projections to confirm the presence of a single root canal and to exclude teeth with anatomical variations. The crowns were sectioned perpendicular to the long axis of the tooth using a water-cooled diamond disc to obtain a standardised root length of 14 mm.

Root canal treatment

Standardised endodontic access cavities were prepared using Endo Access burs. Canal patency was established with a size #10 K-file, and the working length was determined using an electronic apex locator and confirmed radiographically. Root canals were instrumented using the conventional step-back instrumentation technique up to an apical preparation size of ISO #55 K-file.

Throughout biomechanical preparation, the canals were irrigated with 5.25% sodium hypochlorite (HypoChlor Forte, Safe Endo, India), followed by 17% EDTA (EDTA Liquid, WALDENT Innovations Pvt. Ltd., New Delhi, India), and finally flushed with normal saline to remove any residual irrigant. The canals were dried with sterile absorbent paper points and obturated using ISO size #55 gutta-percha cones and AH Plus sealer (Dentsply Sirona, Konstanz, Germany) by the cold lateral compaction technique. All specimens were stored at 37°C and 100% humidity for 24 hours to allow complete setting of the sealer.

Post-space preparation

Post-space preparation was performed after the complete setting of the sealer. Gutta-percha was removed using Peeso reamers (Mani Inc., Tochigi, Japan) in sequential sizes 1, 2, and 3 while maintaining 5 mm of apical gutta-percha to preserve the apical seal, as retaining at least 4-5 mm of root canal filling material has been shown to maintain the integrity of the apical seal following post-space preparation [15]. Post-space preparation was standardised according to the manufacturer's recommendations for the selected glass fibre post system.

Experimental groups

The 40 specimens were randomly allocated into four experimental groups (n = 10 per group) using a computer-generated randomisation sequence. The random allocation sequence was generated before specimen assignment to ensure equal distribution among the four experimental groups. All specimen preparation and fibre post cementation procedures were performed by a single experienced operator using a standardised protocol to minimise procedural variability. Push-out bond strength testing was performed by a second operator who was blinded to the group allocation to minimise measurement bias. In addition, the investigator performing the statistical analysis was blinded to the group allocation to reduce assessment bias. The specimens were assigned to one of the following post-space pretreatment groups: Group I (n = 10): 17% ethylenediaminetetraacetic acid (EDTA); Group II (n = 10): 10% citric acid; Group III (n = 10): 10% polyacrylic acid; and Group IV (n = 10): 37% phosphoric acid.

The conditioning agents used were 17% EDTA (EDTA Liquid, WALDENT Innovations Pvt. Ltd., New Delhi, India), 10% citric acid (CITOCID, AMMDENT India, Mohali, Punjab, India), 10% polyacrylic acid (Crysta Dentin Conditioner, Prevest DenPro Ltd., Jammu, India), and 37% phosphoric acid (Etching Liquid, Prime Dental Products Pvt. Ltd., Mumbai, India).

The concentrations of the conditioning agents were selected based on previous studies demonstrating their effectiveness in smear-layer modification and dentin conditioning before adhesive procedures. A concentration of 17% EDTA was chosen because it is the most widely accepted chelating agent for smear-layer removal in endodontics without causing excessive dentin erosion [7]. Citric acid was used at a concentration of 10%, which has been shown to effectively remove the smear layer while preserving dentin integrity [8]. A 10% concentration of polyacrylic acid was selected because it is commonly recommended as a mild dentin conditioner that partially removes the smear layer while preserving hydroxyapatite for chemical interaction with self-adhesive resin cements [9,16]. Phosphoric acid was used at a concentration of 37% because this concentration is routinely employed for dentin etching and has demonstrated effective smear-layer removal and enhanced resin infiltration in previous studies [12].

Each canal was irrigated with 5 mL of the assigned conditioning agent for 1 minute using a 30-gauge side-vented irrigation needle. The canals were subsequently rinsed with distilled water and dried with sterile paper points before post-cementation.

Fibre post-cementation

Prefabricated glass fibre posts (RelyX™ Fibre Post, 3M ESPE, St. Paul, MN, USA) were cleaned, treated with a silane coupling agent, and cemented according to the manufacturer's recommendations. The posts were luted using RelyX™ U200 self-adhesive resin cement (3M ESPE, St. Paul, MN, USA). The mixed cement was introduced into the prepared post space, and the fibre post was seated to full depth under gentle finger pressure. Excess cement was removed, and polymerisation was carried out according to the manufacturer's instructions. Following cementation, all specimens were stored in distilled water at 37°C for one week before mechanical testing.

Specimen sectioning

Each root specimen was sectioned perpendicular to its long axis using a low-speed diamond saw under continuous water cooling. Three slices, each measuring 2 mm in thickness, were obtained from the coronal, middle, and apical thirds of the root. Slice thickness was verified using a digital Vernier calliper.

Push-out bond strength testing

Push-out bond strength was evaluated using a Universal Testing Machine (50 kN load cell). Each root slice was positioned on the jig in such a manner that the applied load was directed from the apical toward the coronal aspect, thereby ensuring displacement of the fibre post in the direction of least resistance and minimising the influence of canal taper. A cylindrical stainless-steel plunger of appropriate diameter was selected for each root section to ensure that the load was applied exclusively to the fibre post without contacting the surrounding dentin. A compressive load was applied at a crosshead speed of 0.5 mm/min until complete dislodgement of the fibre post occurred. The maximum load required to dislodge the post was recorded in Newtons (N) using the machine software. The push-out bond strength was subsequently calculated by dividing the maximum failure load by the bonded surface area of the post-dentin interface and expressed in Megapascals (MPa) according to the following formula:

{Push-out bond strength (MPa)} = {Failure load (N)} / {Bonded surface area (mm²)}

The bonded surface area was calculated using the formula for the lateral surface area of a truncated cone: 

"$&quot;A=\pi(R+r)\sqrt{h^{2}+(R-r)^{2}}&quot;$" 

where A represents the bonded surface area (mm²), R is the coronal radius of the post, r is the apical radius of the post, and h is the thickness of the root slice (mm). The calculated bond strength values were recorded for the coronal, middle, and apical root sections and subjected to statistical analysis.

Statistical analysis

The collected data were entered into Microsoft Excel (Microsoft Corp., USA) and analysed using GraphPad Prism version 8.4.3 (GraphPad Software, San Diego, CA, USA). Push-out bond strength values were expressed as mean ± standard deviation (SD). Intergroup comparisons among the four post-space pretreatment groups at the coronal, middle, and apical root levels were performed using one-way analysis of variance (ANOVA) followed by Tukey's honestly significant difference (HSD) post-hoc test. Within-group comparisons among the coronal, middle, and apical thirds were performed using a linear mixed-effects model (REML), with tooth treated as a random effect to account for repeated measurements from the same specimen, followed by Tukey's multiple comparisons test. All tests were two-tailed, and a P value < 0.05 was considered statistically significant.

## Results

**Figure 1:**
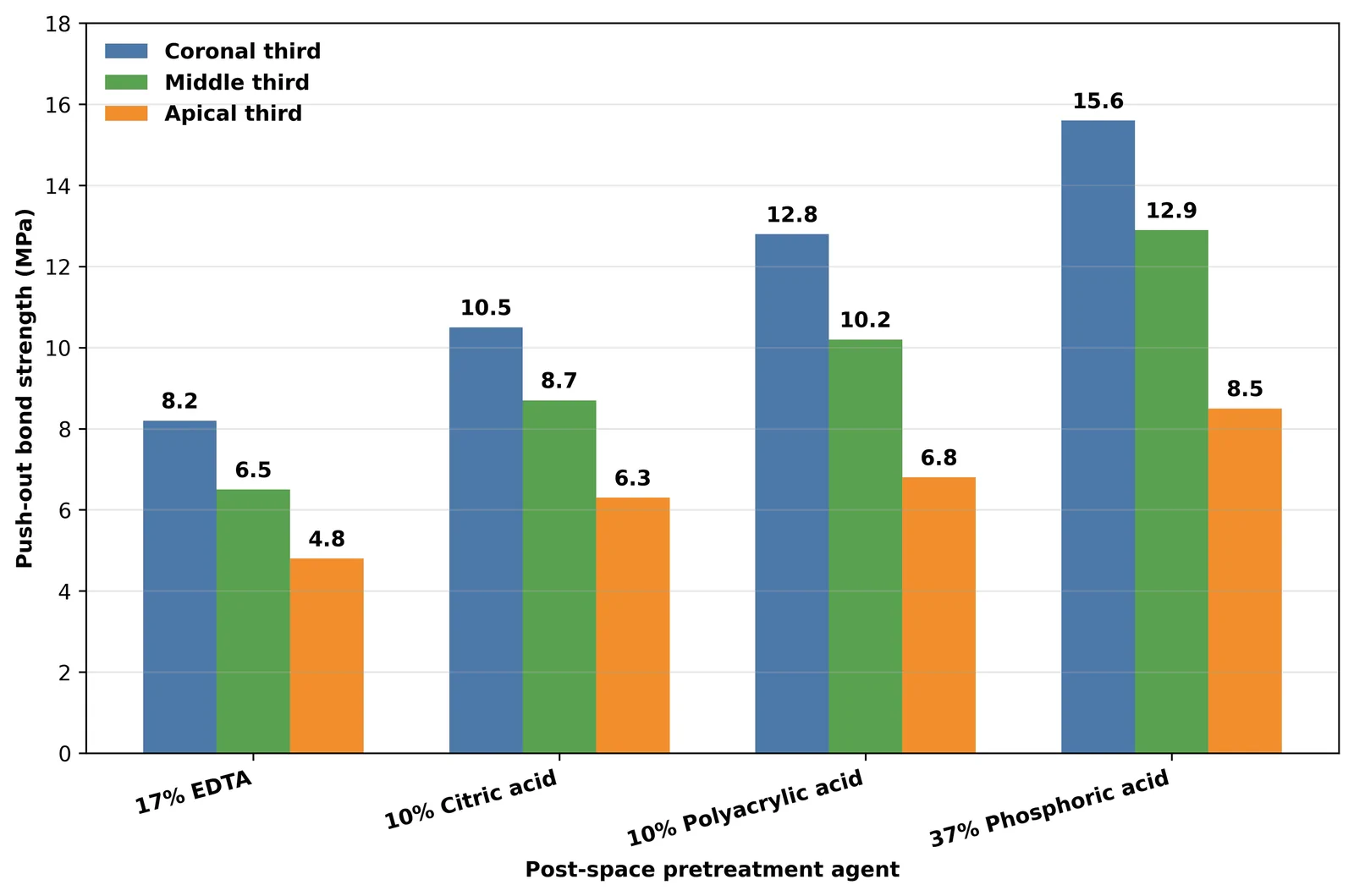
Mean push-out bond strength (MPa) of fibre posts luted with self-adhesive resin cement after different post-space pretreatments.

The mean push-out bond strength values of fibre posts following different post-space pretreatment agents at the coronal, middle, and apical thirds are presented in Table 1 and Figure 1.

**Table 1:** Comparison of push-out bond strength of fibre posts following different post-space pretreatment agents at the coronal, middle, and apical thirds of the root Data are presented as mean ± standard deviation (SD). Intergroup comparisons were performed using one-way analysis of variance (ANOVA). F-statistics are reported for each comparison, followed by corresponding P-values. P < 0.05 was considered statistically significant.

Group	Pretreatment agent	Coronal third (Mean ± SD) (MPa)	Middle third (Mean ± SD) (MPa)	Apical third (Mean ± SD) (MPa)
I	17% EDTA	8.2 ± 1.40	6.5 ± 1.13	4.8 ± 1.04
II	10% Citric acid	10.5 ± 1.60	8.7 ± 1.25	6.3 ± 1.01
III	10% Polyacrylic acid	12.8 ± 1.50	10.2 ± 1.40	6.8 ± 1.13
IV	37% Phosphoric acid	15.6 ± 1.70	12.9 ± 1.50	8.5 ± 1.32
One-way ANOVA (F-value)		41.57	41.02	18.14
P-value		<0.0001	<0.0001	<0.0001

Overall, 37% phosphoric acid produced the highest bond strength at all root levels, followed by 10% polyacrylic acid, 10% citric acid, and 17% EDTA, which consistently showed the lowest values. In all pretreatment groups, bond strength progressively decreased from the coronal to the apical third (Table 1).

Intergroup comparison using one-way ANOVA demonstrated statistically significant differences among the four pretreatment agents at the coronal, middle, and apical thirds (all P < 0.0001) (Table 1). Tukey's post-hoc analysis further showed that most pairwise comparisons were statistically significant. However, no significant difference was observed between the 10% citric acid and 10% polyacrylic acid groups at the middle and apical thirds (Table 2).

**Table 2:** Tukey's HSD post-hoc pairwise comparison of push-out bond strength among different post-space pretreatment agents at the coronal, middle, and apical thirds of the root. Pairwise comparisons were performed using Tukey's honestly significant difference (HSD) post-hoc test following one-way ANOVA. Values of q represent the Studentized range statistic, and P-values are adjusted for multiple comparisons. Adjusted P < 0.05 was considered statistically significant.

Comparison	Coronal third		Middle third		Apical third	
	q	Adjusted P-value	q	Adjusted P-value	q	Adjusted P-value
Group 1 vs. Group 2	4.683	0.0109	5.243	0.0037	4.188	0.0265
Group 1 vs. Group 3	9.366	<0.0001	8.818	<0.0001	5.584	0.0019
Group 1 vs. Group 4	15.070	<0.0001	15.250	<0.0001	10.330	<0.0001
Group 2 vs. Group 3	4.683	0.0109	3.575	0.0724	1.396	0.7576
Group 2 vs. Group 4	10.380	<0.0001	10.010	<0.0001	6.143	0.0006
Group 3 vs. Group 4	5.701	0.0015	6.435	0.0003	4.747	0.0097

Within-group comparisons among the coronal, middle, and apical thirds were performed using a linear mixed-effects model (REML) (Table 3). Significant differences were observed among the three root levels in push-out bond strength among the coronal, middle, and apical thirds for all pretreatment groups (all P < 0.0001).

**Table 3:** Within-group comparison of push-out bond strength among the coronal, middle, and apical thirds using a mixed-effects model (REML) Data are presented as mean ± SD. Comparisons among root levels within each pretreatment group were performed using a linear mixed-effects model (REML). P < 0.05 was considered statistically significant.

Group	Pretreatment agent	Coronal third (Mean ± SD) (MPa)	Middle third (Mean ± SD) (MPa)	Apical third (Mean ± SD) (MPa)	F (DFn, DFd)	P value
I	17% EDTA	8.2 ± 1.40	6.5 ± 1.13	4.8 ± 1.04	F(1.024, 9.220)=172.4	<0.0001
II	10% Citric acid	10.5 ± 1.60	8.7 ± 1.25	6.3 ± 1.01	F(1.091, 9.815)=457.0	<0.0001
III	10% Polyacrylic acid	12.8 ± 1.50	10.2 ± 1.40	6.8 ± 1.13	F(1.369, 12.32)=2126	<0.0001
IV	37% Phosphoric acid	15.6 ± 1.70	12.9 ± 1.50	8.5 ± 1.32	F(1.222, 11.00)=2320	<0.0001

Subsequently, the post-hoc Tukey's multiple comparisons test (Table 4) revealed that all pairwise comparisons among the coronal, middle, and apical thirds were statistically significant within each pretreatment group (all adjusted P values < 0.0001). The greatest mean differences were consistently observed between the coronal and apical thirds, particularly in the 37% phosphoric acid group, whereas the smallest differences occurred between the coronal and middle thirds. Collectively, these results demonstrate a progressive reduction in push-out bond strength from the coronal to the apical region, confirming that both the post-space pretreatment agent and the root level significantly influence the retention of fibre posts.

**Table 4:** Tukey's post -hoc pairwise comparisons of push-out bond strength among the coronal, middle, and apical thirds within each post-space pretreatment group Pairwise comparisons were performed using Tukey's multiple comparisons test following a significant mixed-effects model (REML). Data are presented as mean difference (MPa) with 95% confidence interval (CI). Adjusted P < 0.05 was considered statistically significant.

Group	Pretreatment agent	Comparison	Mean difference	95% CI	P value
I	17% EDTA	Coronal vs. middle	1.70	1.089 to 2.311	<0.0001
		Coronal vs. apical	3.40	2.764 to 4.036	<0.0001
		Middle vs. apical	1.70	1.617 to 1.783	<0.0001
II	10% citric acid	Coronal vs. middle	1.80	1.453–2.147	<0.0001
		Coronal vs. apical	4.20	3.667–4.733	<0.0001
		Middle vs. apical	2.40	2.176–2.624	<0.0001
III	10% polyacrylic acid	Coronal vs. middle	2.60	2.431–2.769	<0.0001
		Coronal vs. apical	6.00	5.674–6.326	<0.0001
		Middle vs. apical	3.40	3.147–3.653	<0.0001
IV	37% phosphoric acid	Coronal vs. middle	2.70	2.412–2.988	<0.0001
		Coronal vs. apical	7.10	6.716–7.484	<0.0001
		Middle vs. apical	4.40	4.231–4.569	<0.0001

## Discussion

The present study evaluated the influence of different post-space pretreatment agents on the push-out bond strength of glass fibre posts luted with self-adhesive resin cement. Post-space conditioning significantly influenced adhesive performance, with 37% phosphoric acid producing the highest bond strength at all root levels, followed by 10% polyacrylic acid, 10% citric acid, and 17% EDTA. These findings emphasise the importance of effective smear-layer modification for achieving durable adhesion between fibre posts, resin cement, and intraradicular dentin.

The superior push-out bond strength observed with 37% phosphoric acid pretreatment may be attributed to its ability to modify or remove the smear layer and facilitate improved resin infiltration into radicular dentin, as reported in previous studies [8]. However, because no SEM or other microscopic evaluation was performed in the present study, these mechanisms remain hypothetical and cannot be directly confirmed by our findings. These findings are consistent with those of Prado et al. [17], who demonstrated superior smear-layer removal with phosphoric acid compared with EDTA and citric acid, as well as Demiryurek et al. [18] and Niu et al. [19], who also reported improved fibre-post bond strength following phosphoric acid conditioning. Together, these studies support phosphoric acid as the most effective conditioning agent for optimising adhesion with self-adhesive resin cements.

The present findings also support the contemporary concepts of adhesive post cementation described by Tsolomitis et al. [6], who emphasised that multiple factors, including post-surface treatment, resin cement selection, irrigants, and cementation protocols, influence fibre-post retention. Our findings extend this concept by demonstrating that effective conditioning of post-space dentin is equally important, as the presence of an intact smear layer may compromise the bonding potential of self-adhesive resin cements.

Among the alternative conditioning agents, 10% polyacrylic acid demonstrated the second-highest bond strength. As a mild conditioner, it partially removes the smear layer while preserving hydroxyapatite necessary for chemical interaction with acidic monomers of self-adhesive resin cements [9]. These findings are consistent with those of Baena et al. [20] and Mazzitelli et al. [16], who also reported improved bonding following polyacrylic acid conditioning. By contrast, 10% citric acid produced intermediate bond strength values. Although effective in smear-layer removal, its greater demineralising effect may reduce the hydroxyapatite available for chemical bonding. Similar observations were reported by Haznedaroğlu [8], explaining why citric acid performed better than EDTA but remained inferior to phosphoric acid.

The lowest bond strength was observed following 17% EDTA pretreatment. Although EDTA effectively removes the inorganic component of the smear layer [7], excessive calcium depletion may reduce chemical interaction between self-adhesive resin cements and dentin [9,14]. This explanation is supported by Faria-e-Silva et al. [12] and Yadav et al. [13], who likewise reported significantly lower bond strength following EDTA pretreatment. These findings suggest that EDTA should be used cautiously as a post-space conditioner when self-adhesive resin cements are employed.

A progressive reduction in bond strength from the coronal to the apical third was observed in all groups. This trend has been attributed to reduced dentinal tubule density, increased dentin sclerosis, limited accessibility of conditioning agents, thicker smear-layer accumulation, and reduced polymerisation efficiency in deeper regions of the root canal [6,21,22]. Similar findings have been reported by Mallmann et al. [21] and Perdigão et al. [22], highlighting the persistent challenge of achieving predictable adhesion in the apical third.

The favourable bond strength observed in the present study may also be attributed to the use of RelyX U200, a dual-cured self-adhesive resin cement. AlMadi et al. [23] demonstrated superior bond strength with dual-cured self-adhesive cement compared with conventional light-cured systems, emphasising the importance of adequate polymerisation throughout the root canal. Nevertheless, our findings indicate that appropriate dentin pretreatment remains essential for maximising the bonding performance of these cements.

Although post-space pretreatment significantly influenced push-out bond strength in the present study, other factors may also have contributed to the observed results. These include the chemical composition and polymerisation characteristics of the self-adhesive resin cement, dentin moisture during cementation, the high configuration factor (C-factor) within the root canal, and adaptation of the fibre post to the prepared post space. These variables may affect resin infiltration, polymerisation stress, and the quality of the adhesive interface and should therefore be considered when interpreting the present findings.

Besides dentin conditioning, other variables also contribute to fibre-post retention. Fernandes et al. [24] highlighted the importance of post adaptation and cement layer thickness, whereas Souza et al. [25] demonstrated that combined physical and chemical post-surface treatments provide superior adhesion. Similarly, Elalfy and Elbasty [26] reported improved bond strength following Er:YAG laser surface treatment. In the present study, all fibre posts received identical silane treatment; therefore, the differences in bond strength can reasonably be attributed primarily to the post-space conditioning protocols rather than variations in post-surface treatment.

Long-term durability of the adhesive interface remains an important consideration. Lima et al. [27] demonstrated improved long-term bond strength with customised fibre posts fabricated using Filtek Z350 composite, whereas Barros et al. [28] reported that post customisation and different LED curing units had minimal influence on immediate bond strength. Although only immediate bond strength was evaluated in the present study, these investigations indicate that ageing, post customisation, and restorative materials may influence long-term clinical performance. Future studies incorporating thermocycling and mechanical fatigue are therefore recommended.

From a clinical perspective, the findings suggest that post-space conditioning before fibre-post cementation can be readily incorporated into routine clinical practice without increasing procedural complexity. Based on the present results, 37% phosphoric acid appears to be the most effective conditioning agent, whereas polyacrylic acid may serve as a suitable alternative when preservation of dentin mineral content is desirable. Conversely, 17% EDTA should be used cautiously because of its adverse effect on bond strength. These observations are consistent with contemporary concepts of adhesive cementation that emphasise optimisation of both dentin and post surfaces for predictable fibre-post retention [6].

The present findings are partly consistent with those of Rajnics et al. [29], who reported that post diameter significantly influenced the push-out bond strength of glass fibre posts, whereas no significant differences were observed among the coronal, middle, and apical root regions. In contrast, the present study demonstrated that post-space pretreatment significantly affected bond strength, with 37% phosphoric acid showing the highest values and the coronal third exhibiting superior bond strength. These differences may be attributed to the different variables evaluated in the two studies.

The findings of the present study have important clinical implications for the restoration of endodontically treated teeth requiring fibre-post rehabilitation. The strengths of this study include standardised specimen preparation, uniform post-space dimensions, computer-generated randomisation, use of a single experienced operator to minimise procedural variability, and evaluation of bond strength at the coronal, middle, and apical thirds, providing a comprehensive assessment of adhesive performance. Collectively, these findings provide clinically relevant evidence to support the selection of an appropriate post-space conditioning protocol for achieving more predictable and durable fibre-post retention. The null hypothesis was rejected, as post-space pretreatment agents significantly influenced the push-out bond strength of glass fibre posts cemented with self-adhesive resin cement.

Limitations of the study

The present study was conducted under standardised in vitro conditions; therefore, the findings may not fully replicate the complex biological, mechanical, and environmental factors encountered in the oral cavity. Only one type of glass fibre post and one self-adhesive resin cement were evaluated, limiting the generalizability of the results to other post systems and luting materials. Furthermore, the effects of thermocycling, mechanical fatigue, long-term ageing, and failure mode analysis were not assessed. In addition, no microscopic evaluation (e.g., SEM) was performed to verify smear-layer removal; therefore, the proposed mechanisms underlying the observed differences in bond strength remain hypothetical. These limitations should be considered when interpreting the findings. Future in vivo studies incorporating different post systems, resin cements, ageing protocols, microscopic evaluation, failure mode analysis, and long-term clinical assessment are warranted to validate the present findings.

## Conclusions

Post-space pretreatment significantly influenced the immediate push-out bond strength of glass fibre posts cemented with self-adhesive resin cement. Among the conditioning agents evaluated, 37% phosphoric acid produced the highest bond strength, followed by 10% polyacrylic acid, 10% citric acid, and 17% EDTA. The mixed-effects analysis also demonstrated significant variations in bond strength among the coronal, middle, and apical root thirds within the experimental groups. These findings indicate that phosphoric acid pretreatment may enhance the immediate adhesion of fibre posts to radicular dentin when used with self-adhesive resin cement. However, as the present investigation evaluated only immediate laboratory bond strength under controlled in vitro conditions, the findings should be interpreted with caution. Further studies incorporating ageing protocols and well-designed clinical trials are warranted to confirm the long-term clinical applicability of these results.
